# Are You a Friend or an Enemy? The Dual Action of Methylglyoxal on Brain Microvascular Endothelial Cells

**DOI:** 10.3390/ijms26115104

**Published:** 2025-05-26

**Authors:** Roberta Moisă (Stoica), Călin Mircea Rusu, Antonia Teona Deftu, Mihaela Bacalum, Mihai Radu, Beatrice Mihaela Radu

**Affiliations:** 1 Department of Anatomy, Animal Physiology and Biophysics, Faculty of Biology, University of Bucharest, 050095 Bucharest, Romania; roberta.stoica@nipne.ro (R.M.); calinmircea@gmail.com (C.M.R.); teona.deftu@yahoo.com (A.T.D.); beatrice.radu@bio.unibuc.ro (B.M.R.); 2 Department of Life and Environmental Physics, Horia Hulubei National Institute for Physics and Nuclear Engineering, 077125 Măgurele, Romania; bmihaela@nipne.ro

**Keywords:** methylglyoxal, blood–brain barrier, calcium mobilization, cytoskeleton, barrier permeabilization

## Abstract

Methylglyoxal is a reactive dicarbonyl intermediate in the advanced glycation end-product (AGE) pathway, and alterations in its levels have been detected in the plasma, cerebrospinal fluid, and brain parenchyma in various pathologies, particularly in diabetes. In this study, we investigate the effects of methylglyoxal (MGO) on murine brain microvascular endothelial cells at both physiological and pathological concentrations. We evaluate molecular parameters, including reactive oxygen species (ROS) production, cytosolic calcium signaling, and ATP synthesis, as well as cellular responses such as cytoskeletal remodeling, cell migration, adhesion, and permeability, across a concentration range of 0–1000 μM. At low concentrations (below ~250 μM), MGO does not induce oxidative stress; instead, it leads to an increase in cytosolic calcium levels and ATP production. At higher concentrations, however, MGO induces significant oxidative stress, which is accompanied by a marked decrease in cell viability, particularly at concentrations exceeding 500 μM. The modulation of key functional processes, including purinergic calcium signaling, actin filament synthesis, cell migration, and adhesion, reveals a threshold concentration beyond which cellular function is impaired due to oxidative stress. Below this threshold, the observed effects appear to be mediated primarily by non-oxidative mechanisms, likely involving protein glycation. In conclusion, our results suggest a dual action of methylglyoxal on brain endothelial cells, with distinct molecular mechanisms underlying its effects at physiological versus pathological concentrations.

## 1. Introduction

Methylglyoxal (MGO) is an α-dicarbonyl compound formed through different biochemical processes from pyruvic acid. In addition to its enzymatic pathway, MGO can also be formed spontaneously during glycolysis, from dihydroxyacetone phosphate as a by-product of 3-phosphate-glyceraldehyde formation in most cells, including endothelial cells [1,2]. Glycolysis is not a completely harmless process to cells, as it inevitably leads to the production of MGO as an auxiliary metabolite. Its accumulation in cells has major adverse effects, as it is one of the most potent intracellular glycation agents. Methylglyoxal reacts rapidly with proteins, lipids, and nucleic acids, leading to the formation of advanced glycation end-products (AGEs). Under normal physiological conditions, the detoxification of MGO is primarily mediated by the glyoxalase enzymatic system, which maintains its intracellular concentration at low, non-toxic levels [3,4,5]. However, in cases where the glyoxalase system is impaired, MGO accumulates to higher levels, potentially exerting harmful effects on cellular function and viability.

MGO has been extensively described as a key molecule in diabetes and its complications and is increasingly recognized as an emerging diagnostic biomarker [4,5,6,7,8,9]. Moreover, MGO is believed to link the underlying mechanisms of diabetes and cancer and has been identified as a pro-tumorigenic molecule [10]. For instance, multiple studies have demonstrated that patients with diabetes exhibit a higher risk of developing cancer [11].

A substantial body of research has investigated the role of MGO in neurological disorders. MGO has been shown to contribute to oxidative stress, playing a role in the onset of Alzheimer’s disease, particularly due to impairments in its detoxification via the glutathione-dependent glyoxalase system [12,13]. This observation aligns with the well-established fact that diabetes is a risk factor for the development of Alzheimer’s disease and other forms of dementia [14]. In addition, MGO has been implicated in several other neurological conditions. It has been shown to influence the onset of Parkinson’s disease [15], modulate seizure susceptibility in experimental models of epilepsy [16], and exacerbate ischemia-induced cerebral vascular degeneration [17,18]. All these neurodegenerative and neuroinflammatory disorders share a common feature: the functional disruption of the neurovascular unit (NVU), which often involves increased permeability of the blood–brain barrier (BBB) [19,20,21,22].

Additionally, MGO has been shown to disrupt the glycolytic metabolic balance within the NVU, which comprises a complex interplay between neurons, astrocytes, and endothelial cells [23]. Astrocytes utilize extracellular glucose and serve as a primary source of lactate for neurons [24]. Within this metabolic triangle, brain endothelial cells play a key role in regulating lactate bioavailability in the brain parenchyma [25], primarily by metabolizing glucose into lactate [26].

In this context, it has been demonstrated that MGO induces BBB damage and increased permeability [27]. The proposed mechanisms involve the accumulation of AGEs, which disrupt the mitochondrial oxidative balance, leading to multiple downstream effects: (i) functional disturbances in cellular metabolism, including the dysregulation of proteins involved in tight junction formation (e.g., occludin and zona occludens-1) [28], and (ii) the upregulation of AGE receptors (RAGE) along with increased expression of pro-inflammatory interleukins IL-6 and IL-8 [28,29,30,31]. Furthermore, AGEs formed through the exposure of basement membrane proteins, such as fibronectin and laminin, to MGO have been shown to reduce the adhesion capacity of retinal microvascular endothelial cells [32] and increase the permeability of human brain microvascular endothelial cell (HBMEC) monolayers [31].

Given the critical role of the BBB in the processes described above, the aim of our study is to uncover additional aspects of the complex mechanisms through which MGO acts on brain endothelial cells and consequently affects the functionality of the NVU. Considering the two most prominent primary processes triggered by MGO, namely protein glycation and the generation of reactive oxygen species (ROS), as reported in the literature, we evaluate the influence of MGO on a set of cellular and molecular processes in brain microvascular endothelial cell cultures, which serve as a simplified in vitro model of the BBB. These include the following: (i) cytotoxic effects, such as alterations in cell proliferation; (ii) molecular effects, including mitochondrial and total ROS production, ATP synthesis, and actin cytoskeleton reorganization; and (iii) functional effects, such as monolayer permeability and its recovery following injury, endothelial adhesion capacity, and modifications in calcium signaling. Some of these biological endpoints were used to confirm the well-established effects of MGO, such as cytotoxicity, ROS production, and changes in permeability. Others, to the best of our knowledge, were investigated for the first time in brain microvascular endothelial cells, including ATP production, the quantitative analysis of cytoskeletal remodeling, and cytosolic calcium signaling. In our previous work on the effects of MGO in cultured neurons [33], we observed a dual influence, with distinct manifestations depending on MGO concentration. In the present study, we investigate the concentration-dependent effects of MGO on the aforementioned biological endpoints, aiming to identify potential nonlinear responses.

## 2. Results

### 2.1. Methylglyoxal Inhibits Cell Proliferation and Stimulates Mitochondrial ROS Production in Brain Microvascular Endothelial Cells

**Figure 1:**
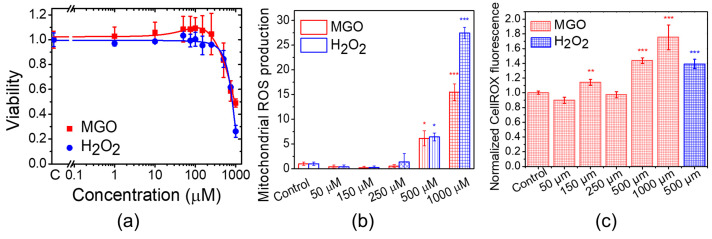
MGO induces dose-dependent changes in cell viability, mitochondrial ROS production, and total ROS production in bEnd.3 cells. (**a**) Cell viability after 24 h of exposure to MGO. (**b**) Mitochondrial ROS production after 24 h of exposure to MGO. (**c**) Total intracellular ROS production after 24 h of exposure to MGO. Data are presented as the mean ± SD (n = 3). Statistical analysis was performed using one-way ANOVA followed by the Fisher post hoc test. Statistical significance is indicated as follows: * *p* < 0.05, ** *p* < 0.01, and *** *p* < 0.001.

To evaluate the effect of MGO on cell viability and proliferation, we measured MTT reduction to formazan crystals in bEnd.3 cells 24 h after exposure (Figure 1a). This reduction reflects the number of metabolically active cells [34]. We observed a significant decrease in proliferation at MGO concentrations above 500 μM, as indicated by reduced MTT activity. H_2_O_2_ caused a more pronounced reduction in MTT activity at similar concentrations. However, MGO (1–250 μM) slightly stimulated cell viability, though the differences were not statistically significant. Viability values for MGO concentrations between 10 and 250 μM were higher than control values, whereas H_2_O_2_ showed a plateau in viability across the same concentration range. These results suggest distinct, dose-dependent effects of MGO and H_2_O_2_ on cell proliferation, which are likely due to different molecular mechanisms. Although MGO (1–250 μM) did not affect cell viability, we examined whether it induced oxidative stress. We measured mitochondrial ROS production (Figure 1b) and total intracellular ROS (Figure 1c). At concentrations of 500 and 1000 μM MGO, ROS levels significantly increased, primarily from the mitochondria, suggesting impaired mitochondrial function. Similarly, H_2_O_2_ induced oxidative stress in a dose-dependent manner, with the strongest effect at 1 mM.

### 2.2. Methylglyoxal Modulates the Equilibrium of Free Cytosolic Calcium Ions in Brain Endothelial Cells

Although calcium plays a physiological role in supporting mitochondrial function and ATP synthesis, at elevated concentrations, it can stimulate the production of ROS, ultimately leading to apoptosis. This delicate interplay—often referred to as the Ca^2+^–ATP–ROS triangle—maintains a dynamic equilibrium that finely balances non-toxic, homeostatic conditions with the risk of mitochondrial dysfunction [35]. To test the hypothesis that MGO modulates the interplay between ROS generation, cytosolic calcium ion dynamics, and ATP production, we first investigated the effects of MGO on cytosolic calcium levels under both basal (equilibrium) and stimulated conditions.

**Figure 2:**
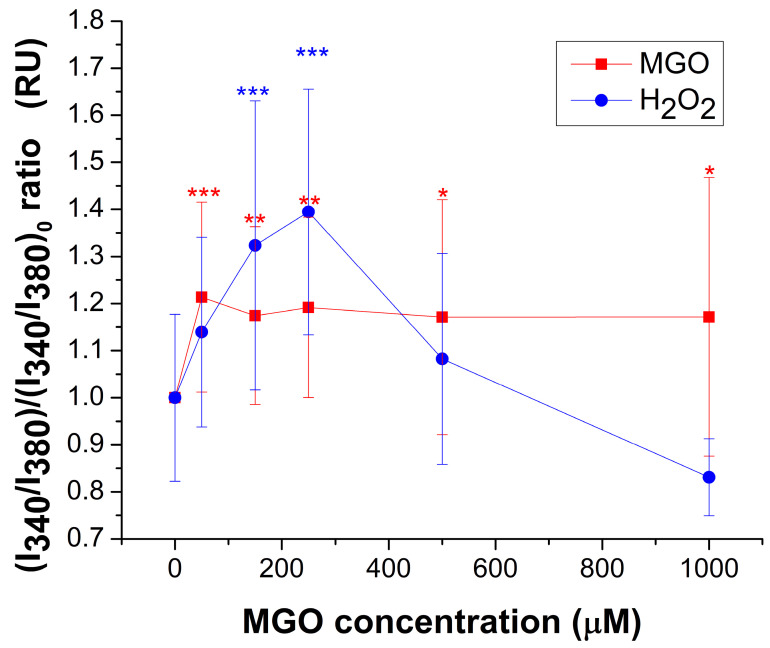
Relative change (mean ± SD) of basal cytosolic Ca^2+^ concentration, expressed as the I_340_/I_380_ ratio, in response to varying concentrations of MGO or H_2_O_2_. At least 25 cells were recorded for each condition. Statistical analysis was performed with one-way ANOVA followed by the Fisher post hoc test. Statistical significance is indicated as follows: * *p* < 0.05, ** *p* < 0.01, and *** *p* < 0.001.

MGO induces a modest but statistically significant increase (~20%) in basal free cytosolic Ca^2+^ levels across the entire concentration range tested (Figure 2). In contrast, H_2_O_2_ markedly increased cytosolic calcium levels within the 10–250 μM range, reaching a maximum of approximately 40% above the control. At higher concentrations, calcium levels returned to baseline, most likely due to cellular damage caused by oxidative stress. These findings clearly indicate that MGO and H_2_O_2_ exert their effects through distinct mechanisms, particularly at lower concentrations.

### 2.3. MGO Upregulates ATP Release, Exhibiting a Window-like Dose–Response Pattern

**Figure 3:**
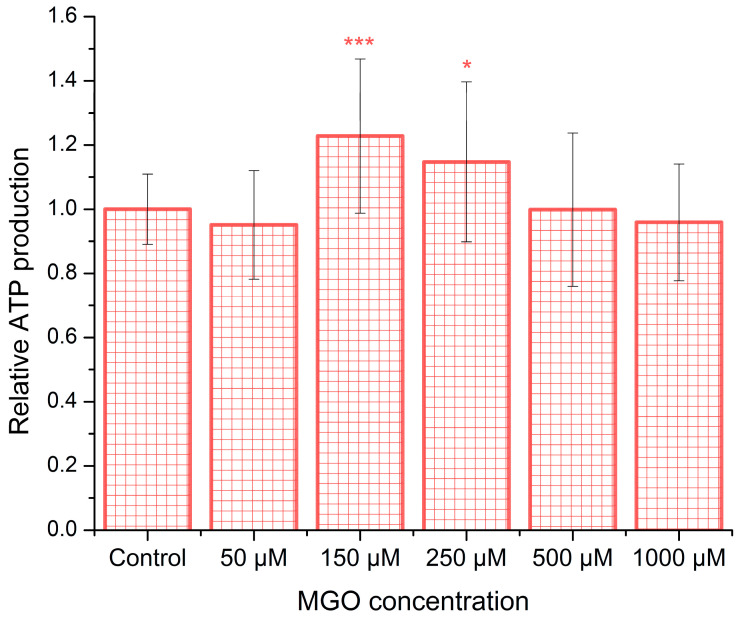
MGO induces dose-dependent alterations in ATP release. ATP levels released from bEnd.3 cells after 12 h of MGO exposure are shown as the mean ± SD from five independent experiments. Statistical analysis was conducted using one-way ANOVA followed by the Bonferroni post hoc test. Significance levels are indicated as follows: * *p* < 0.05 and *** *p* < 0.001.

To investigate the influence of methylglyoxal (MGO) on ATP synthesis, brain endothelial cells were exposed to various concentrations of MGO for 12 h. As shown in Figure 3, ATP production has a statistically significant increase in the range of 150–250 μM MGO compared to control conditions, with the maximal increase being ~23%. However, at the higher MGO concentration, ATP production recovers to the control values, suggesting a window-like dose–response. To the best of our knowledge, there is currently no published evidence regarding ATP release from bEnd.3 cells or from any type of brain microvascular endothelial cells in the presence of MGO.

### 2.4. Methylglyoxal Modulates ATP-Induced Cytosolic Calcium Transients in Brain Endothelial Cells

**Figure 4:**
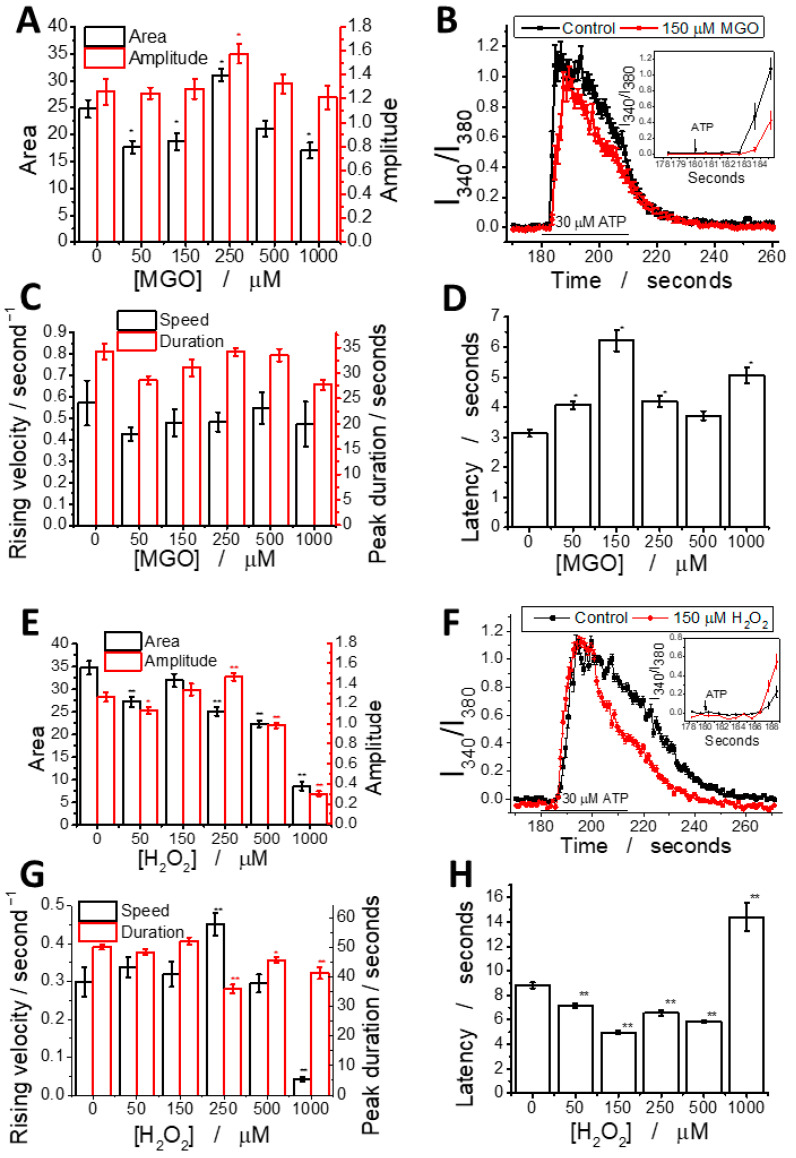
MGO alters ATP-induced calcium signal parameters in brain microvascular endothelial cells. MGO (**A**–**D**) modulates calcium signaling in a manner distinct from H_2_O_2_ (**E**–**H**). Graphs show calcium signal parameters: amplitude and area (**A**,**E**), rising velocity and duration (**C**,**G**), and latency (**D**,**H**) as a function of concentration; representative ATP-induced calcium peaks at 150 μM MGO (**B**) or H_2_O_2_ (**F**) compared to the control, with latency differences highlighted in the inset. Additional parameters are provided in the Supplementary Materials. Statistical significance was assessed using one-way ANOVA (at least 25 cells have been recorded for each experimental condition), followed by the Fisher post hoc test. The statistical significance is indicated as follows: * *p* < 0.05 and ** *p* < 0.01.

In addition to affecting basal Ca^2+^ levels, MGO modulates the shape of ATP-induced Ca^2+^ transients in brain endothelial cells in a dose-dependent manner following 24 h of exposure (Figure 4). A detailed analysis revealed that MGO has a statistically significant impact on multiple parameters of the ATP-induced calcium signal, including the area under the curve, amplitude, latency, duration, and rising velocity, as calculated using previously established methods [36]. For comparison, the effect of H_2_O_2_ across the same concentration range was also evaluated.

We analyzed the effects of MGO (0–1000 μM) on ATP-induced calcium signaling (Figure 4A–D). The calcium peak area showed a biphasic response: a 25% decrease at 50–150 μM, a 20% increase at 250 μM, and a decline at higher concentrations. Peak amplitude increased by ~20% at 250 μM, indicating a threshold effect. Latency was significantly increased (~100%) at 150 μM, while other parameters showed no significant changes. Rising velocity and duration followed similar patterns, with a Pearson correlation coefficient of 0.75. This is the first report showing that MGO modulates calcium signaling in brain microvascular endothelial cells.

For comparison, H_2_O_2_ (Figure 4E–H) reduced the peak area at all concentrations, with the highest concentration decreasing it to 20% of the control. Peak amplitude showed a slight increase up to 250 μM, followed by a sharp drop. Latency decreased in most conditions, except at the highest concentration where it nearly doubled. Rising speed remained similar to the control, except at 250 μM (increase) and 1000 μM (dramatic decrease). The duration remained constant up to 250 μM, then decreased slightly at higher concentrations.

We observed a strong positive correlation between the peak area, amplitude, and duration, while rising velocity was positively correlated with the duration and asymmetry, likely due to the coupling between the initial calcium release and store-operated calcium entry (SOCE). A negative correlation between latency and other parameters suggests that delayed signal transduction reduces the rising velocity, asymmetry, and duration. These findings indicate the dysregulation of IP_3_-dependent signaling and SOCE mechanisms. Pearson correlation coefficients for each pair of parameters were also calculated (Figures S2–S7 and Tables S1 and S2). In H_2_O_2_-treated cells, the correlation pattern looks different, suggesting that MGO and H_2_O_2_ act through distinct mechanisms, despite both generating ROS (Table S3).

To determine the source of ATP-induced calcium transients in our experiments, we used AR-C 118925XX, a selective P2Y_2_ receptor antagonist previously shown to be effective in bEnd.3 cell cultures [37]. To further investigate the role of IP_3_ receptors, calcium transients were measured in a calcium-free extracellular solution. As expected, both the amplitude and area of the calcium signals were significantly reduced in the presence of the inhibitor (Figure S10). Notably, the latency of the transients increased approximately threefold, suggesting that AR-C 118925XX exerts a complex modulatory effect on P2Y_2_ receptor signaling. Interestingly, treatment with 150 μM MGO partially restored calcium transient parameters toward control levels, although this compensatory effect was less pronounced at 250 μM MGO.

### 2.5. Methylglyoxal Induces Cytoskeletal Reorganization in Brain Microvascular Endothelial Cells, Leading to Impaired Migration and Increased Monolayer Permeability

**Figure 5:**
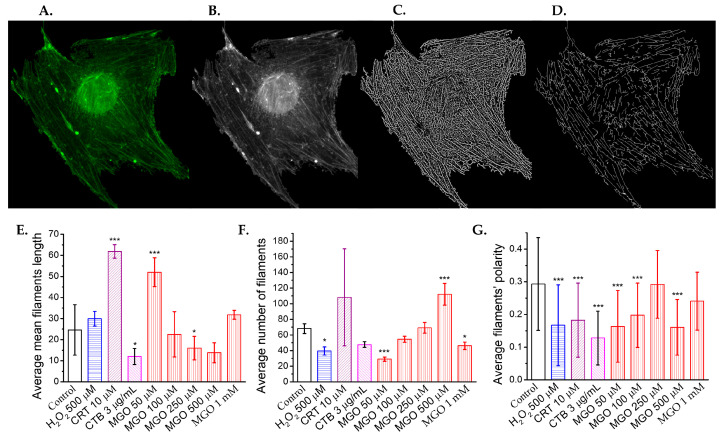
Methylglyoxal remodels the cytoskeleton of brain microvascular endothelial cells. (**A**) Representative confocal microscopy images of actin filaments stained with phalloidin-FITC. (**B**) Grayscale-converted confocal images used as the input for the Fiberscore algorithm. (**C**) Fiber index images obtained after applying the convolution mask. (**D**) Thinned fiber skeletons generated following the application of the thinning mask (scale bar: 10 μm). Effects of methylglyoxal and positive cytoskeletal remodeling controls (H_2_O_2_, 500 μM; cytochalasin B [CTB], 3 μg/mL; and cofilin inhibitor CRT0105950, 10 μM) on (**E**) the average filament length, (**F**) the average number of filaments, and (**G**) the filament’s polarity, as determined from confocal images using the Fiberscore algorithm. (A total of 57 cells have been analyzed.) Statistical significance was assessed using one-way ANOVA, followed by the Bonferroni post hoc test, and is indicated as follows: * *p* < 0.05 and *** *p* < 0.001.

To evaluate the effect of methylglyoxal (MGO) on actin filaments in the endothelial cell cytoskeleton, confocal microscopy images of cells with fluorescently labeled actin filaments were acquired (see examples in Figure S8). The images were digitally processed using the Fiberscore algorithm [38]. Representative images from each step of the analysis were selected, including the original confocal microscopy images, grayscale-converted images, fiber index maps generated after applying the convolution mask, and thinned fiber skeletons obtained via the thinning mask (Figure 5). Subsequently, the mean values of key parameters, such as filament length, filament number, and filament polarity, were extracted using the Fiberscore algorithm and compared across different treatment conditions. For the proper assessment of MGO-induced changes in actin filament organization, three positive controls were used: hydrogen peroxide, cytochalasin D (known to disrupt actin filaments through distinct mechanisms) [39], and CRT 0105950, a potent inhibitor of cofilin phosphorylation [40].

MGO significantly affects all analyzed filament parameters (Figure S1). The mean actin filament length increases more than twofold at 50 μM MGO, similar to the effect of CRT 0105950, but decreases with higher MGO concentrations, reaching levels comparable to CTB at 500 μM (Figure 5E). In contrast, the average number of filaments decreases with increasing MGO concentrations, consistent with the positive controls (Figure 5F). The increase in filament number at lower concentrations, coupled with a decrease in filament length, suggests actin fragmentation.

Actin filament polarity significantly decreases at 50 and 100 μM MGO (*p* < 0.001), showing values similar to those with H_2_O_2_ and CRT 0105950, but higher than CTB. Polarity returns to control levels at 250 μM, then decreases again at 500 μM (Figure 5G). Histograms of cell number versus polarity (Figure S9) show a narrowed distribution at 50 and 100 μM, a slight broadening at 250 μM, and a modest increase at 1000 μM, likely due to intensified oxidative stress, which causes actin to form hay-like bundles. However, interpreting parameters at 1000 μM MGO is challenging due to the low signal-to-noise ratio and difficulty in accurately segmenting filaments.

**Figure 6:**
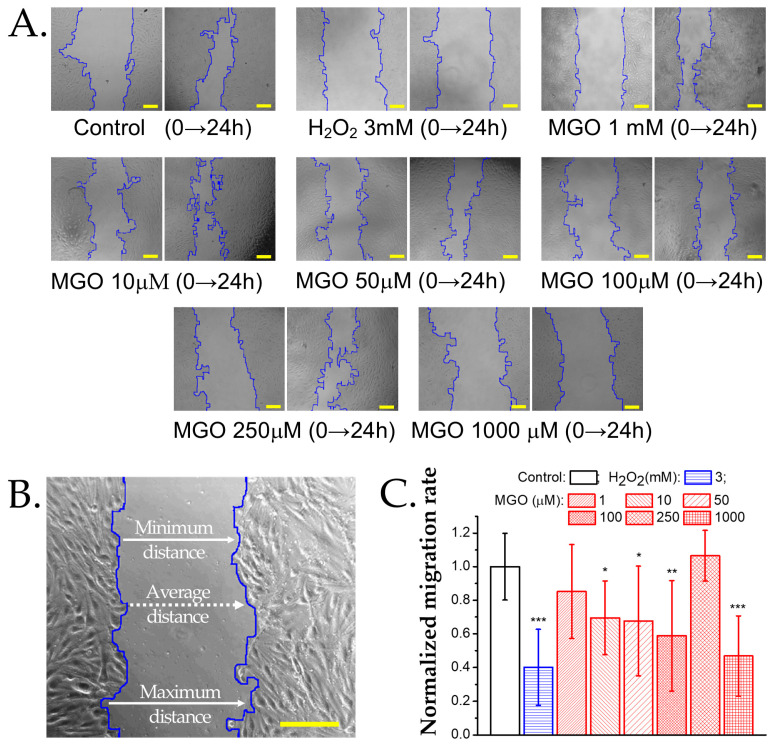
Effect of methylglyoxal on the migration rate of brain microvascular endothelial cells. (**A**) Transmitted light microscopy images of wound healing assays at baseline (0 h) and after 24 h of exposure to MGO or the positive control H_2_O_2_. Scale bars: 30 μm. (**B**) Wound width measured along the edges of the cellular region in a representative sample of brain microvascular endothelial cells. For details on the analysis algorithm, refer to the Materials and Methods Section. Scale bar: 30 μm. (**C**) Normalized migration rate (mean ± SD, n at least 7) after 24 h of exposure to H_2_O_2_ (positive control) or MGO (1, 10, 50, 100, 250, 1000 μM). Statistical analysis was performed using one-way ANOVA, followed by the Bonferroni post hoc test. Statistical significance is indicated as follows: * *p* < 0.05, ** *p* < 0.01, and *** *p* < 0.001.

To assess cell migration following an injury, we performed wound healing assays. Low MGO concentrations (1 μM) slightly reduced the migration rate, though not significantly compared to the controls (Figure 6). At 50 μM and 100 μM, the migration rate decreased by approximately 50%, and at 1000 μM, migration was strongly inhibited, similar to the effect of H_2_O_2_ (3 mM). Interestingly, at 250 μM MGO, migration was unaffected and comparable to the controls.

**Figure 7:**
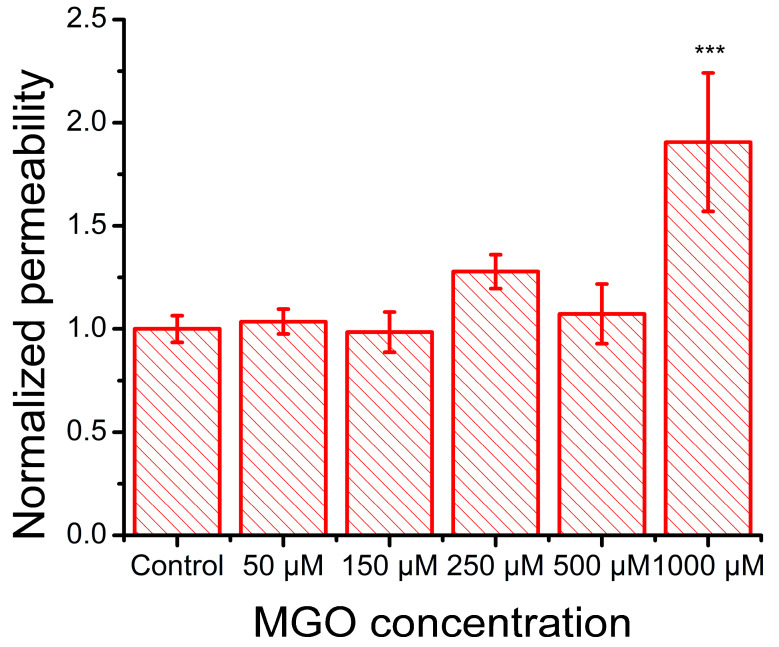
Effect of MGO on the FITC–Dextran 4000 permeability of an in vitro monolayer of cultured brain microvascular endothelial cells (mean ± SD, n = 3). Statistical analysis was conducted using one-way ANOVA, followed by Bonferroni post hoc comparisons. Statistical significance is indicated as follows: *** *p* < 0.001.

Previous studies have demonstrated that methylglyoxal downregulates the expression of tight junction proteins, including claudin-5, ZO-1, and occluding, in bEnd.3 cells [28]. In our experiments, FITC permeability assays revealed that the treatment of endothelial cell monolayers with 50 μM and 150 μM methylglyoxal had no observable effect on barrier integrity. However, exposure to 250 μM MGO showed a tendency toward increased permeability, and only the 1000 μM concentration resulted in a significant disruption of monolayer integrity (Figure 7).

### 2.6. MGO Enhances Leukocyte Adhesion to Brain Microvascular Endothelial Cells

**Figure 8:**
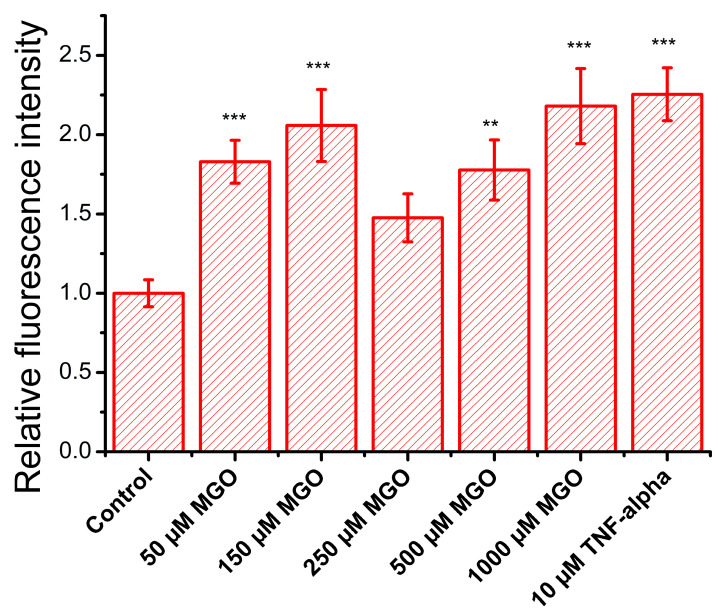
Effect of MGO on the relative adhesion rate (relative fluorescence intensity, mean ± SD, n = 3) of a lymphocyte cell model (calcein-labeled Jurkat cells) to a brain microvascular endothelial cell monolayer. Statistical analysis was conducted using one-way ANOVA, followed by Fisher’s post hoc test. Statistical significance is indicated as follows: ** *p* < 0.01, and *** *p* < 0.001.

During oxidative stress, endothelial cells produce elevated ROS, which upregulate the expression of adhesion molecules, facilitating immune cell recruitment, vascular permeability, and their transmigration into the subendothelial space, contributing to inflammation onset [41]. To assess the impact of MGO on these processes, we evaluated the expression of adhesion molecules in brain microvascular endothelial cells. TNF-α, a pro-inflammatory cytokine that activates NF-κB, was used as a positive control, and as expected, it doubled the adhesion capacity of endothelial cells (Figure 8), confirming assay reliability. As anticipated, MGO, due to its pro-oxidant properties, increased Jurkat cell adhesion, suggesting the upregulation of endothelial adhesion molecules. This effect followed a nonlinear dose–response pattern, with two concentration ranges showing pronounced effects: (i) low concentrations (50 and 150 μM) significantly increased adhesion, reaching levels similar to TNF-α, and (ii) high concentrations (500 and 1000 μM) also produced a comparable effect. At 250 μM, adhesion returned to baseline, with no significant difference from the control. These findings suggest that MGO modulates endothelial function through multiple concentration-dependent mechanisms, likely involving distinct signaling pathways or cellular stress responses.

## 3. Discussion

While our research specifically explores the nuanced effects of MGO on the BBB, it is important to note that many prior studies examining MGO’s impact on brain microvascular endothelial cells have utilized substantially higher concentrations, most commonly around 2 mM [18] and in some cases as high as 10 mM [17,30]. Additionally, these investigations have been conducted primarily on human brain endothelial cells, whereas murine brain microvascular endothelial models remain comparatively underrepresented in the literature.

Our findings on MGO-induced impairment of cell viability are consistent with the results reported by Kim et al. [28], who observed a significant reduction in cell viability at concentrations above 500 μM. Notably, Kim and colleagues also reported a slight increase (~5%) in cell proliferation following 24 h of exposure to 100 μM MGO (Figure 1F in [28]), which is in line with our observations, although in both cases, the effect was not statistically significant. The interpretation of MGO’s impact on endothelial cell viability remains complex, as the literature presents divergent findings that range from stimulatory effects to no effect or even cytotoxicity at comparable concentrations [27,28,42,43]. These inconsistencies may stem from variations in endothelial cell type, species origin, cell density, or other experimental conditions used across studies.

We observed that MGO-induced production of reactive oxygen species (ROS)—both mitochondrial and cytosolic—increased significantly at concentrations above 250 μM. The similarities between MGO and H_2_O_2_ in their effects on cell viability and ROS generation suggest that the inhibition of cell proliferation by MGO is likely mediated through oxidative stress pathways in endothelial cells [44]. Direct quantitative comparisons with other studies remain challenging due to differences in experimental conditions. However, our results are qualitatively consistent with those by Kim et al., who reported a 1.5-fold increase in cytosolic ROS after 9 h and a ~5-fold increase in mitochondrial ROS after 24 h of MGO exposure, although the specific MGO concentration was not disclosed [28]. The degree of ROS production varies substantially across cell types and experimental models. For example, Dhar et al. reported a 2-fold increase in ROS in rat aortic endothelial cells and a 1.5-fold increase in human umbilical vein endothelial cells after exposure to just 30 μM MGO, though cell viability was not assessed in that study [45]. Similarly, Alomar et al. observed a ~4-fold increase in mitochondrial ROS after 24 h of treatment with 30 μM MGO in primary human brain endothelial cells, along with a ~50% reduction in cell viability at 40 μM [46]. Together, these findings reinforce the link between elevated ROS production and reduced cell viability, although the threshold MGO concentrations at which this relationship becomes evident appear to be cell type-dependent.

The interrelationship between cytosolic calcium levels, energy metabolism, and redox balance plays a critical role in both cellular physiology and pathology [35]. Under normal conditions, moderate increases in free cytosolic calcium stimulate mitochondrial ATP synthesis [35]. In our experiments, we observed an approximate 20% elevation in basal cytosolic calcium levels across all concentrations of MGO, which may partially explain the increased ATP production observed at 150–250 μM MGO. This effect could be attributed to MGO-induced glycation of proteins involved in calcium regulation. A similar mechanism has been described in β-cells, where MGO-mediated glycation led to a reduction in SERCA pump activity [36]. At higher MGO concentrations, however, ATP production declined, likely as a result of excessive oxidative stress affecting both mitochondrial and cytosolic compartments. In contrast, H_2_O_2_ exposure produced a strong increase in basal calcium levels (~140%) at low concentrations, followed by a sharp decline above 300 μM, presumably due to oxidative damage—a pattern consistent with previously reported findings [47,48]. Moreover, since vascular endothelial cells predominantly rely on glycolysis for ATP production [49], our observation of an approximately 23% increase in ATP levels following treatment with 150 μM MGO is consistent with previous findings reporting a ~20% enhancement of glycolytic activity in aortic endothelial cells treated with 100 μM MGO [50]. A related mechanism has also been described, linking ATP production to basal cytosolic calcium levels under stress conditions. This involves the activation of mechanosensors in human umbilical and rat aortic endothelial cells but, notably, not in human embryonic kidney (HEK293T) cells [51].

Our analysis of the calcium–ATP–ROS axis indicates that methylglyoxal (MGO) exerts a dual effect on brain endothelial cells. At low concentrations, MGO promotes metabolic stimulation, likely through cytosolic calcium elevation, which enhances ATP production. In contrast, higher concentrations of MGO induce oxidative stress, disrupting cellular homeostasis. Notably, 250 μM MGO appears to represent a critical threshold that delineates the transition between these opposing effects. This biphasic response is consistent with our previous findings in cultured mouse neurons, where a similar dual action of MGO was observed [33]. In the following sections, we further investigated this working hypothesis by examining more complex cellular processes. The functional outcomes evaluated in this study, including ATP-induced cytosolic calcium transients, barrier permeability, and cell adhesion and migration, offer additional evidence for the concentration-dependent dual effects of MGO on brain microvascular endothelial cells.

Pro-angiogenic stimuli (e.g., ATP, ADP, and acetylcholine) activate diverse Ca^2+^ responses via IP_3_ receptors and SOCE through Orai1 channels [52]. Evidence suggests that purinergic receptor sensitivity is reduced under hyperglycemic conditions [53]. Notably, MGO has been shown to modulate the activity of various ion channels: it inhibits the voltage-gated sodium channel Nav1.8 [54] and the transient receptor potential melastatin 8 (TRPM8) channel [55] while activating the human transient receptor potential ankyrin 1 (TRPA1) channel [37] and store-operated calcium entry (SOCE) pathways [56]. Since bEnd.3 cells express P2Y2 purinergic receptors [57], we hypothesized that MGO may modulate purinergic signaling pathway activity in our samples. The data show the window-like, dose-dependent modulation of ATP-induced calcium transients by MGO: an amplitude and area peak at 250 μM and latency peaks at 150 μM. In contrast, H_2_O_2_ showed a minimal effect beyond a slight enhancement in rising velocity, with most changes occurring within the concentration range where ROS production was observed. We propose that this biphasic response reflects a shift from protein glycation at lower MGO concentrations (affecting GPCRs, IP_3_ receptors, and SOCE proteins) to oxidative stress at higher levels. Given the reported calcium dependence of IP_3_ signaling [58] and glycation-induced IP_3_ receptor impairment [59], the increase in latency likely indicates reduced IP_3_ receptor activity. Conversely, oxidative stress alone, which is induced by H_2_O_2_, only shortened the latency (Figure 4).

The partial inhibition of calcium transients by AR-C 118925XX, a selective P2Y_2_ receptor antagonist, suggests that additional P2Y receptor subtypes may be expressed in bEnd.3 cells. This interpretation is partly supported by the only existing study using AR-C 118925XX in bEnd.3 cultures [57], which reported a substantially stronger inhibitory effect—approximately 6-fold—compared to the ~2.5-fold reduction observed in our experiments. The expression of multiple P2Y receptor subtypes across various endothelial cell types has also been documented [60,61], reinforcing the notion of a complex purinergic signaling network. Interestingly, the partial restoration of calcium transient parameters in cells treated with 150 μM MGO suggests that MGO may mitigate receptor inhibition in a concentration-dependent manner. At 250 μM MGO, this compensatory effect is less evident, consistent with our earlier observation of the calcium transient amplitude and area peak at MGO concentrations between 150 and 250 μM.

Cytoskeletal remodeling is essential for cellular adaptation to microenvironmental cues and is tightly regulated by actin polymerization, a process in which calcium ions play a key role [62,63]. In our study, MGO-induced elevation of cytosolic calcium likely promotes actin filament formation through calcium-sensitive proteins such as cofilin [64]. This is supported by similar effects observed with CRT 0105950, a LIM kinase inhibitor, which mimics 50 μM MGO by increasing filament length (Figure 5E). At higher MGO concentrations, oxidative stress predominates, leading to actin fragmentation, consistent with previous studies [65,66]. Our results show a positive correlation between MGO concentration and filament fragmentation (Figure 5F). Unlike H_2_O_2_ and cytochalasin B (CTB), which reduce both filament length and number, MGO at 500 μM appears to simultaneously promote filament formation via calcium and fragmentation via oxidative stress, resulting in a high filament count. These structural changes correspond with previously observed morphological alterations such as reduced cell area and increased optical thickness [27], while polarity scores (Figure 5G) further suggest a dual MGO effect, with 250 μM as a functional threshold.

Although actin polymerization does not require ATP hydrolysis, ATP influences filament dynamics and rigidity, with ATP-bound filaments being more stable [67]. Thus, changes in ATP levels directly impact cytoskeletal remodeling. The ATP increase observed at 150–250 μM MGO may support actin polarity restoration. Endothelial cell migration, such as in wound healing assays, is closely linked to actin remodeling [68]. Notably, both the migration rate and actin polarity recover at 250 μM MGO. A recent study showed that migration through dense collagen increases the ATP:ADP ratio, while easier migration reduces it [69]. Since ROS levels remain low at <500 μM MGO, non-oxidative mechanisms like actin glycation [70] may explain early cytoskeletal depolarization and impaired migration. At ≥500 μM MGO, oxidative stress dominates, leading to cell death, depolarization, and reduced actin fiber density, ultimately suppressing migration.

MGO has been shown to compromise the blood–brain and blood–retinal barriers by reducing tight junction proteins (e.g., occluding and claudin-5) and activating MMP-2/9 in human endothelial cells [27,29]. In bEnd.3 cells, we observed similar effects: high MGO concentrations led to increased barrier permeability, is strongly correlated with MGO-induced ROS, and is likely linked to AGE formation. Using the same cell line, Kim et al. [28] also reported that MGO triggers mitochondrial oxidative stress, affecting tight junction integrity. While in vivo studies have shown that MGO upregulates adhesion molecules via ROS signaling [71,72,73,74], in vitro evidence is limited, and to our knowledge, bEnd.3 cells have not previously been used in this context [74,75,76]. Using a Jurkat cell adhesion assay, we observed a biphasic response: increased adhesion at low and high MGO concentrations, with no significant effect at 250 μM. This suggests the involvement of distinct mechanisms, such as ROS-driven mechanisms at high concentrations and potentially glycation-mediated mechanisms at lower levels. Indeed, several studies have reported the involvement of advanced glycation end-products (AGEs) and their receptors in mediating the cellular effects of MGO. Protein glycation induced by 0.1–1 mM MGO has been shown to upregulate AGE receptor expression in human brain endothelial cell cultures, with subsequent impacts on barrier function [31]. A limitation of our study is the absence of the direct quantification of AGEs and their receptors, which prevents definitive conclusions regarding their involvement. However, previous findings [31] lend support to our hypothesis that MGO-induced glycation processes may contribute to the effects observed at low MGO concentrations.

## 4. Materials and Methods

### 4.1. Brain Endothelial Cells

Mouse brain microvascular endothelial cells (bEnd.3, ATCC, ^®^ CRL-2299™, Manassas, VA, USA) were used in this study. Cells were cultured in Advanced DMEM (Gibco, 12491023, New York, NY, USA) supplemented with 10% fetal bovine serum (Gibco, 11570506), 1% L-glutamine (Biowest, X0550, Nuaillé, France), and 100 U/mL penicillin with 100 μg/mL streptomycin (Gibco, 15140-122), as previously described [77]. Cell density varied depending on the experiment. Twenty-four hours after plating, cells were treated for an additional 24 h with one of the following: methylglyoxal (MGO; Sigma-Aldrich, #67028, Darmstadt, Germany), a potent glycation agent known to induce oxidative stress; hydrogen peroxide (H_2_O_2_; Sigma-Aldrich, #216763, Darmstadt, Germany), which was used as a positive control for oxidative stress; and cytochalasin B (CTB; Sigma-Aldrich, #C6762, Darmstadt, Germany), which was used as a positive control for cytoskeletal remodeling, as it is a well-characterized, cell-permeable mycotoxin that inhibits actin filament network formation.

### 4.2. Cell Viability Assay

For this assay, 10,000 cells were seeded into each well of a 96-well plate. After 24 h of treatment, endothelial cell proliferation was assessed using the MTT assay (#20395, Serva, Heidelberg, Germany), applied at a final concentration of 0.5 mg/mL, and incubated for 3 h. MGO was tested at concentrations ranging from 1 to 1000 μM. Optical density was measured at 570 nm using a Mithras ELISA plate reader (Berthold Technologies, Bad Wildbad, Germany). Cell viability was calculated relative to untreated controls, and all measurements were performed in triplicate.

### 4.3. Mitochondrial and Total ROS Production

For the assessment of mitochondrial ROS production, 10,000 cells were seeded per well in 96-well plates. For each experiment, at least three wells per condition were analyzed. Following MGO exposure, mitochondrial reactive oxygen species were measured using MitoSOX™ Red (#M36008, ThermoFisher, Waltham, MA, USA), a fluorescent probe specific for mitochondrial superoxide, following the manufacturer’s protocol. ROS values for each condition were normalized to the untreated control. Results are expressed as relative values. Total intracellular ROS was measured using the CellROX™ Green assay (C10444, ThermoFisher, Waltham, MA, USA), in accordance with the manufacturer’s instructions. Cells (~30,000 per coverslip) were seeded on 10 mm diameter coverslips. After MGO exposure, cells were incubated with the CellROX reagent for 30 min at 37 °C, fixed with 4% paraformaldehyde (PFA), permeabilized with 0.5% Triton X-100, and stained with Hoechst 33342 to visualize nuclei. For each condition, at least 10 fluorescence images per sample were acquired. The mean fluorescence intensity per cell was calculated by dividing the total fluorescence intensity by the number of nuclei.

### 4.4. ATP Production

To evaluate changes in ATP production in treated cells, we performed ATP quantification using a bioluminescence assay kit based on recombinant firefly luciferase and D-luciferin (A22066, ThermoFisher, Waltham, MA, USA), following the manufacturer’s protocol. All experiments were conducted in triplicate, with 10,000 cells seeded per sample. Results are presented as ATP levels relative to control conditions.

### 4.5. Calcium Imaging and Calcium-Signal Parameter Analysis

Ratiometric calcium imaging was used to assess calcium ion dynamics in brain microvascular endothelial cells under both control and oxidative stress conditions, as previously described [36]. Cells were incubated with 1 μM Fura-2 acetoxymethyl ester (F1221, ThermoFisher, Waltham, MA, USA), a dual-wavelength, calcium-sensitive fluorescent dye, along with 0.1% Pluronic F-127 (Life Technologies, Carlsbad, CA, USA), at room temperature in the dark for 45 min. Samples were maintained in Ringer’s solution containing (in mM) 140 NaCl, 5.6 KCl, 2 MgCl_2_, 2 CaCl_2_, 10 glucose, and 10 HEPES, adjusted to a final pH of 7.4. For the inhibition experiments, endothelial cells were maintained in a calcium-free (0 mM Ca^2+^) Ringer solution containing (in mM) 140 NaCl, 5.6 KCl, 2 MgCl_2_, 10 HEPES, and 16 glucose, adjusted to a final pH of 7.4.

Calcium measurements were performed using a cooled CCD camera (Andor iXon EM, Oxford Instruments, Abingdon, United Kingdom) and a Polychrome V monochromator (Till Photonics GmbH, Grafelfing, Germany) coupled to an Olympus IX71 inverted fluorescence microscope equipped with a 40× water immersion objective (Olympus Corporation, Tokyo, Japan). Data acquisition was conducted using the Andor iQ 1.8 software, and changes in intracellular calcium concentration were determined by calculating the fluorescence intensity ratio (I_340_/I_380_). Ratios were computed for each individual cell within the microscope field using manually defined regions of interest, following background subtraction. Solutions were applied to the brain endothelial cells via a 100 μm quartz perfusion head, which was connected to an eight-channel pressurized valve system (ALA Scientific Instruments). Cytosolic calcium transients triggered by the activation of purinergic receptors were elicited by a 30 s pulse of 30 μM ATP. To assess the behavior of the BBB model under oxidative stress conditions, cells were pre-exposed for 24 h to varying concentrations of MGO (50–1000 μM). For cells treated with 10 μM AR-C, samples were incubated at room temperature for 10 min prior to calcium transient recordings. Calcium transients were then induced by a 5 min application of 30 μM extracellular ATP.

The shape of the calcium signals was analyzed using several parameters: amplitude (the maximum value of the transient), duration (the time from stimulus application to return to baseline), area (the integral under the transient curve, which is dependent on both amplitude and duration), latency (the delay between stimulus application and the onset of the calcium transient), rising velocity (the absolute value of the maximum first derivative of the signal), and asymmetry (calculated as b/a, where a is the time from response onset to peak, while b is the time from peak to return). These parameters were extracted using a MATLAB R2020a-based program previously developed [38] and adapted for the conditions of our experiments. Additionally, Pearson correlation coefficients were calculated for each pair of calcium signal parameters.

### 4.6. Adhesion Assay

We investigated whether the expression of adhesion molecules on the surface of endothelial cells is modulated by methylglyoxal (MGO) treatment. To this end, we prepared a bEnd.3 cell monolayer, which was treated with MGO concentrations ranging from 50 to 1000 μM, as well as with 10 ng/mL TNF-α as a positive control [78]. After 24 h of treatment, 25,000 Jurkat cells (TIB-152, ATCC, Manassas, VA, USA), which were labeled with 2 μM Calcein Green AM (C34852, Thermo Fisher, Waltham, MA, USA), were added to each sample. Following a 2 h incubation period and several PBS washes, fluorescence images were acquired using an Olympus BX-51 epifluorescence microscope (Olympus Corporation, Tokyo, Japan). To quantify Jurkat cell adhesion under each condition, fluorescence intensity was measured using the ImageJ 1.43u software. Data were reported as the ratio of the average intensity for each treatment condition over the average intensity of the control.

### 4.7. Permeabilization Assay

Endothelial cells were cultured on Transwell Permeable Support Inserts (3470, Costar Corning, New York, NY, USA) until they reached confluency. The cells were then treated for 24 h with MGO at concentrations ranging from 50 to 1000 μM. To assess the degree of permeabilization of the blood–brain barrier (BBB) model following MGO treatment, 200 μg/mL FITC-conjugated dextran, with an average molecular weight of 4000 (46944, Sigma-Aldrich), was added to the apical chamber of the insert. Culture media were collected from the basolateral chamber at 15, 30, and 45 min. The fluorescence of the media was measured using an ELISA plate reader (Mithras, Berthold Technologies, Bad Wildbad, Germany). The permeability coefficient was calculated and normalized with respect to the control values.

### 4.8. Wound Healing Assay

To assess the basal cell migration ability of endothelial cells, confluent monolayers were serum-starved for 6 h, after which a wound was created in the monolayer using a P10 pipette tip. Cells were visualized at baseline and 24 h after exposure to MGO or H_2_O_2_ (3 mM) using an inverted microscope (Nikon, Tokyo, Japan) with a 20× objective. Images were captured using the uEye Cockpit software. Each condition was performed in triplicate, and the experiment was repeated twice on different cell passages.

The wound width was calculated using a previously described algorithm [79]. This algorithm detected the boundaries of the cellular regions on each side of the “wound” using frequency filtering and mathematical morphology methods, independent of wound orientation and under conditions of high cell sparsity. The cell migration rate was calculated as previously described [80], and the normalized migration rate for each treatment condition was reported relative to the control.

### 4.9. Fluorescent Staining of Actin Filaments and Confocal Image Acquisition

A total of 30,000 bEnd.3 cells per sample were exposed for 24 h to various concentrations of MGO or to the positive controls such as H_2_O_2_ (3 mM), CTB (3 μg/mL), and the cofilin inhibitor CRT 0105950 (10 μM). For staining, cells were washed three times with PBS (5 min each), fixed for 5 min with 3% paraformaldehyde (#158127-100g, Sigma-Aldrich), and then washed again with PBS. Cells were permeabilized with 0.1% Triton X-100 in PBS, followed by additional PBS washes. Cytoskeletal actin filaments were stained with 20 μg/mL phalloidin–FITC (#ab235137, Abcam, Cambridge, United Kingdom) at room temperature for 1 h, and nuclei were stained with 2 μg/mL Hoechst 33342 (#H3570, Invitrogen, Waltham, MA, USA). Finally, cells were mounted on FluorSave™ glass slides (Merck Millipore, Burlington, MA, USA).

Fluorescence images of actin filaments were acquired using an Andor DSD2 confocal unit ( Oxford Instruments, Abingdon, United Kingdom) mounted on an Olympus BX-51 epifluorescence microscope (Olympus Corporation, Tokyo, Japan) equipped with a 40× objective and appropriate filters for GFP/FITC (466/40 nm excitation, 488 nm dichroic mirror, and 525/54 nm emission) and DAPI (390/40 nm excitation, 405 nm dichroic mirror, and 452/45 nm emission).

### 4.10. Confocal Image Processing for Cytoskeletal Changes

For the confocal image analysis, the cells were manually individualized using Fi-ji/ImageJ in order to avoid unwanted detection during further template-based segmentation. We employed an adapted MATLAB implementation of the Fiberscore algorithm developed by Nurit Lichtenstein for fiber pattern identification, taking advantage of its robustness against low signal-to-noise ratios and unevenly distributed intensities [38].

Actin filament detection was performed by correlating pixel neighborhood regions in the confocal microscopy images with corresponding linear pixelated templates oriented in various directions. This template-based convolution step was followed by the application of a thinning mask, which reduced the detected filaments to their characteristic morphological skeleton [36]. For quantitative cytoskeletal analysis, the thinning mask was binarized to eliminate dependence on pixel intensity. Subsequent analysis involved quantifying the number of detected actin filaments and calculating the mean fiber length per cell. Cell polarity, representing the degree of filament parallelism, was calculated using the following formula:

Polarity = √(D_x_^2^ + D_y_^2^)

where D_x_ and D_y_ represent the cosine and sine components, respectively, along the best-correlated linear structures aligned with the filaments in the image [36]. Polarity distributions were analyzed based on their tails and full width at half maximum (FWHM).

To determine the optimal parameter set for the Fiberscore algorithm, we conducted an in-depth analysis of its performance under varying conditions, including a comparison with the original parameters proposed by Lichtenstein et al. We examined contour plots to identify values in the parameter space that yielded the highest number of detected filaments within a minimum fiber length range of 5 to 30 μm (Supplementary Figure S1). This analysis focused on the following parameters: M (threshold for the normalized standard deviation along a linear filament structure), N (threshold for the ratio of normalized standard deviations between filament structures), TC (threshold for the correlation coefficient between pixelated linear structures and actual actin filaments), and T (threshold for image pixel intensity) [38]. Parameter optimization was based on the confocal microscopy images of bEnd.3 cells under control conditions to tailor the settings to the specific morphological characteristics of this cell line. The computational parameters used in our implementation of the Fiberscore algorithm are listed in Table 1. It has been previously observed that variations in the L (convolution kernel matrix size) and K (angular resolution of the pixelated fiber structure) parameters, as shown in Table 1, can lead to the over- or under-segmentation of confocal microscopy images [81].

This quantitative approach to actin filament analysis is robust against common imperfections in fluorescence microscopy images, which are often encountered in both fixed and live-cell imaging. It effectively addresses challenges such as variable cell thickness, which can cause defocusing; uneven background fluorescence (arising from nonspecific labeling, autofluorescence, or unassembled cytoplasmic monomers); and low signal-to-noise ratios. These issues can be mitigated through careful parameter selection [38,81]. At least 50 cells were analyzed for each condition.

### 4.11. Statistical Analysis

Data were plotted using OriginPro 8 (OriginLab Corporation, Northampton, MA, USA) and are presented as the mean ± standard deviation (SD). Statistical significance between treatment conditions (MGO or H_2_O_2_ at various concentrations) and the control was assessed using one-way ANOVA, followed by the Bonferroni post hoc test. Significance levels were indicated as follows: * *p* < 0.05, ** *p* < 0.01, and *** *p* < 0.001. All experiments were performed in triplicate and repeated at least three times for each experimental technique.

## 5. Conclusions

This study reveals new insights into how MGO impacts the brain microvascular endothelium, highlighting its role in regulating ATP production, actin cytoskeleton organization, endothelial repair after injury, cytosolic calcium homeostasis, and ATP-induced calcium signaling. By combining our results on ATP levels, intracellular calcium dynamics, and ROS generation, we suggest that MGO exerts a dual, concentration-dependent effect on brain endothelial cells. The functional outcomes we observed, together with the existing literature, could support this hypothesis; however, future studies have to be performed to definitely prove the proposed hypothesis. These findings emphasize the importance of considering the brain endothelium as an active component of the neurovascular unit and suggest that its dysregulation under oxidative stress may play a significant role in the development of various neurological disorders.

## Figures and Tables

**Table 1:** Computational parameters used for the application of the Fiberscore algorithm.

Parameters	Value
Kernel matrix size (L)	6
Angular resolution (K)	10
Threshold correlation coefficient (TC)	0.55
Threshold for the normalized standard deviation (M)	0.3
Threshold for the ratio of the normalized standard deviations (N)	2.1
Threshold pixel intensity (T)	0.25

## Data Availability

The original contributions presented in this study are included in the article; further inquiries can be directed to the corresponding author.

## Supplementary Materials

The following supporting information can be downloaded at https://www.mdpi.com/article/10.3390/ijms26115104/s1 [82].
