# Supplementary material for: Are You a Friend or an Enemy? The Dual Action of Methylglyoxal on Brain Microvascular Endothelial Cells
Source: Int J Mol Sci. 2025 May 26;26(11):5104. doi: 10.3390/ijms26115104 (PMC12154078; doi:10.3390/ijms26115104)
Supplement: Supplementary file 1 [file ijms-26-05104-s001.zip › ijms-3612744-supplementary.pdf]

International Journal of
Molecular Sciences

MDPI

Supplementary Material

Are You a Friend or an Enemy? The Dual Action of Methylglyoxal on Brain Microvascular Endothelial Cells

Roberta Moisă (Stoica) [1,2], Călin Mircea Rusu [1], Antonia Teona Deftu [1], Mihaela Bacalum [2], Mihai Radu [2,*] and Beatrice Mihaela Radu [1]

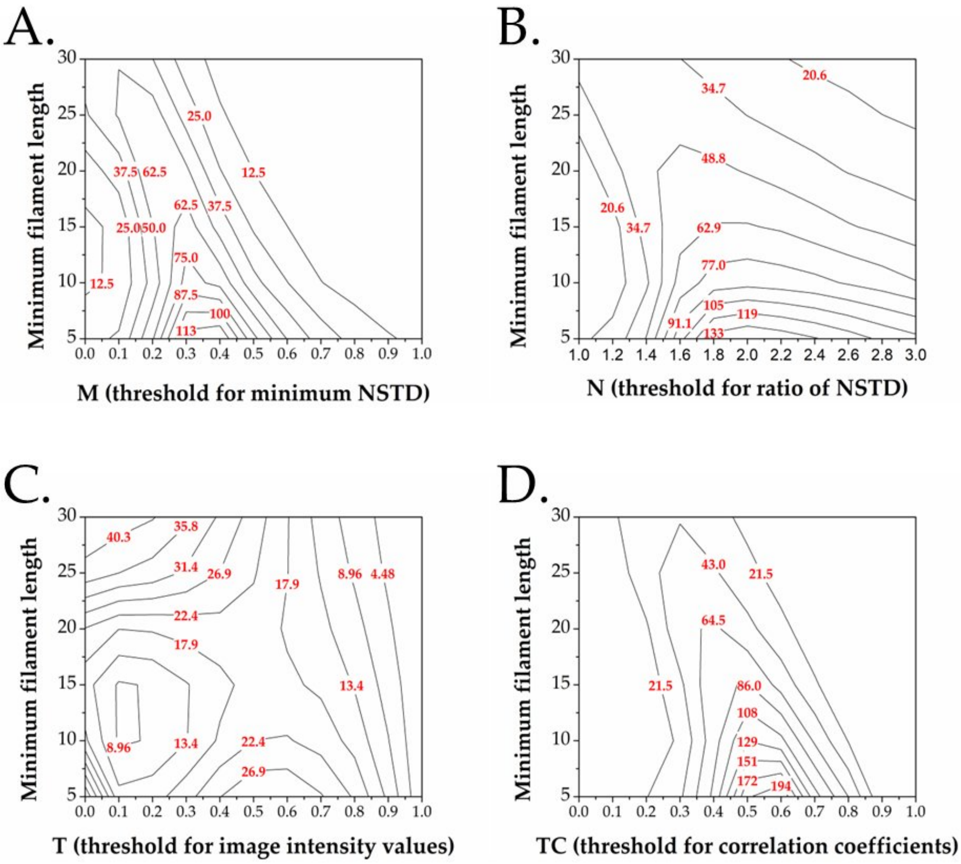

Figure S1. Parameter contour plots using confocal microscopy images of bEnd.3 cells under control conditions: (a) M: Threshold for the minimum normalized standard deviation along the linear template. (b) N: Threshold for the ratio of normalized standard deviations between two linear templates. (c) T: Threshold for pixel intensity values. (d) TC: Threshold for accepted correlation coefficients. The y-axis of the contour plots represents the minimum accepted filament length, the x-axis shows the value of the varied parameter along the parameter space, and the colormap indicates the average number of fibers above the length specified on the y-axis.

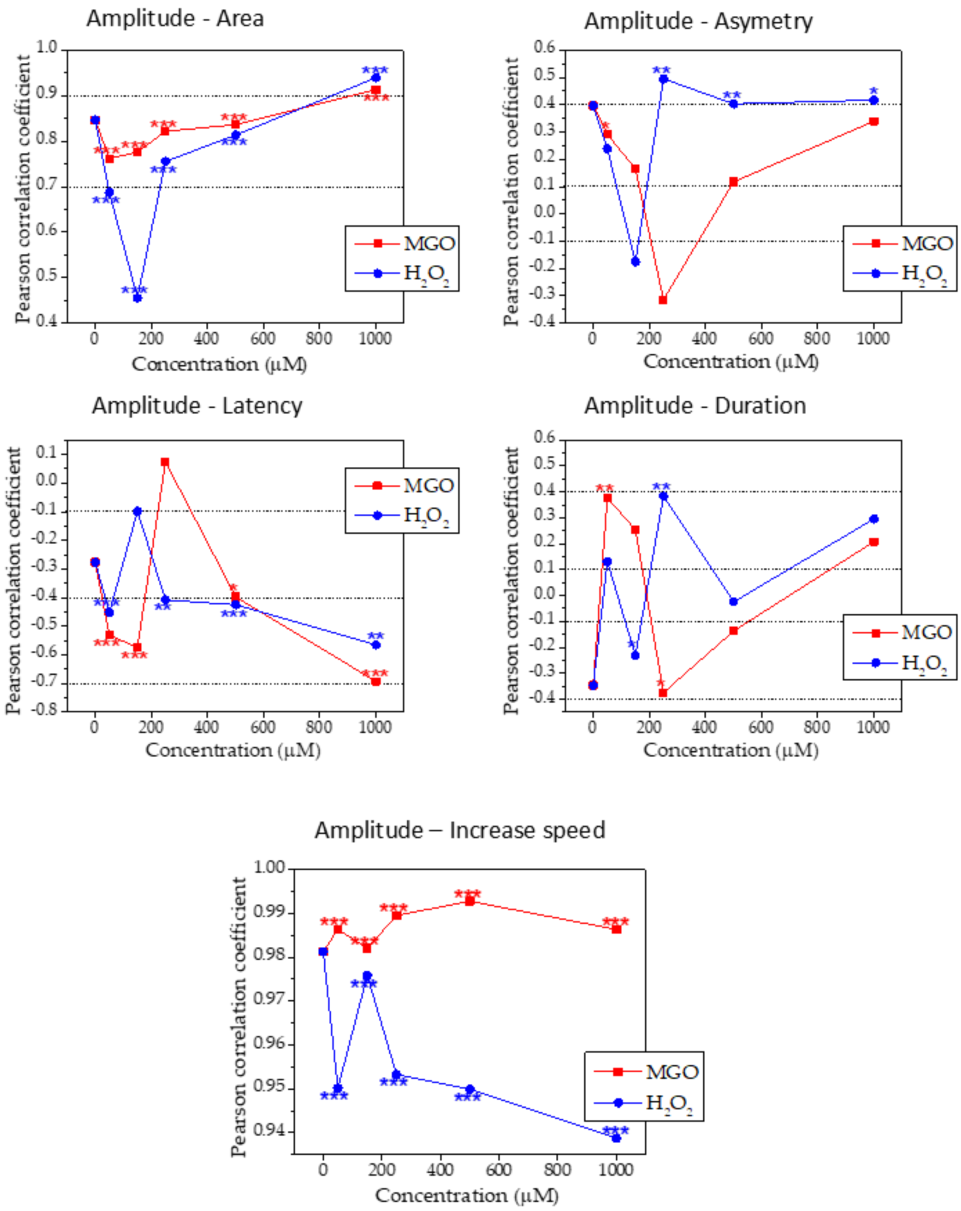

Figure S2. Pearson correlation coefficients between the amplitude and the other parameters of the ATP-induced calcium signals. Brain microvascular endothelial cells were exposed for 24-h to different concentrations of MGO (50, 150, 250, 500, 1000 µM) or H_2O_2 (50, 150, 250, 500, 1000 µM). Statistical analysis was done by one-way ANOVA followed by *post-hoc* Bonferroni test, and indicated as * $p<0.05$, ** $p<0.01$, *** $p<0.001$. Pearson correlation coefficients between amplitude and other parameters of the ATP-induced calcium signals. Brain microvascular endothelial cells were exposed for 24 hours to various concentrations of MGO (50, 150, 250, 500, 1000 µM) or H_2O_2 (50, 150, 250, 500, 1000 µM). Statistical analysis was performed using one-way ANOVA, followed by the *post-hoc* Bonferroni test, with significance indicated as $p < 0.05$, $p < 0.01$, *$p < 0.001$.

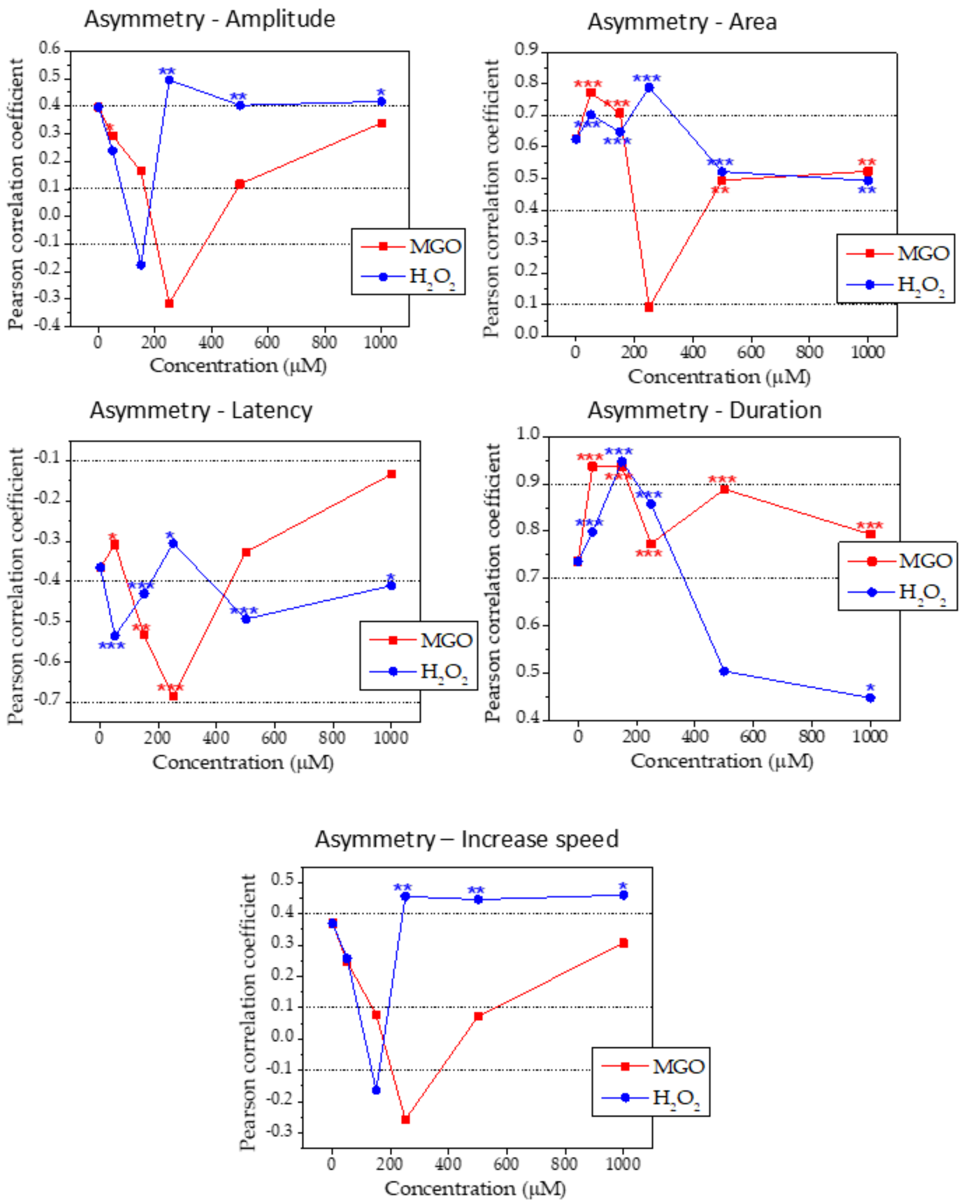

Figure S3. Pearson correlation coefficients between the asymmetry and other parameters of the ATP-induced calcium signals. Brain microvascular endothelial cells were exposed for 24 hours to various concentrations of MGO (50, 150, 250, 500, 1000 μM) or H2O2 (50, 150, 250, 500, 1000 μM). Statistical analysis was performed using one-way ANOVA, followed by the *post-hoc* Bonferroni test, with significance indicated as * p<0.05, ** p<0.01, *** p<0.001.

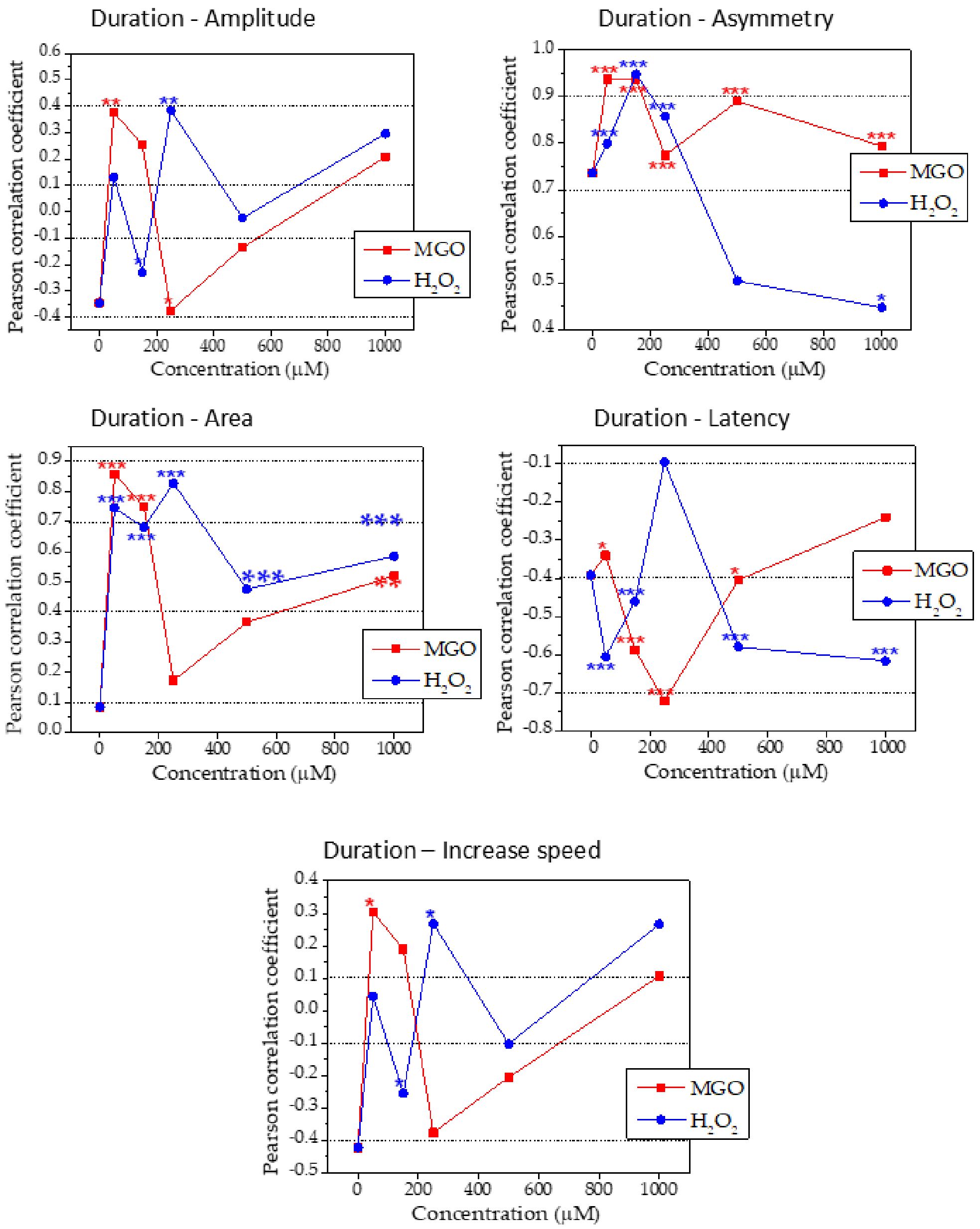

Figure S4. Pearson correlation coefficients between duration and other parameters of the ATP-induced calcium signals. Brain microvascular endothelial cells were exposed for 24 hours to various concentrations of MGO (50, 150, 250, 500, 1000 µM) or H_2O_2 (50, 150, 250, 500, 1000 µM). Statistical analysis was performed using one-way ANOVA, followed by the *post-hoc* Bonferroni test, with significance indicated as $p < 0.05$, $p < 0.01$, *$p < 0.001$.

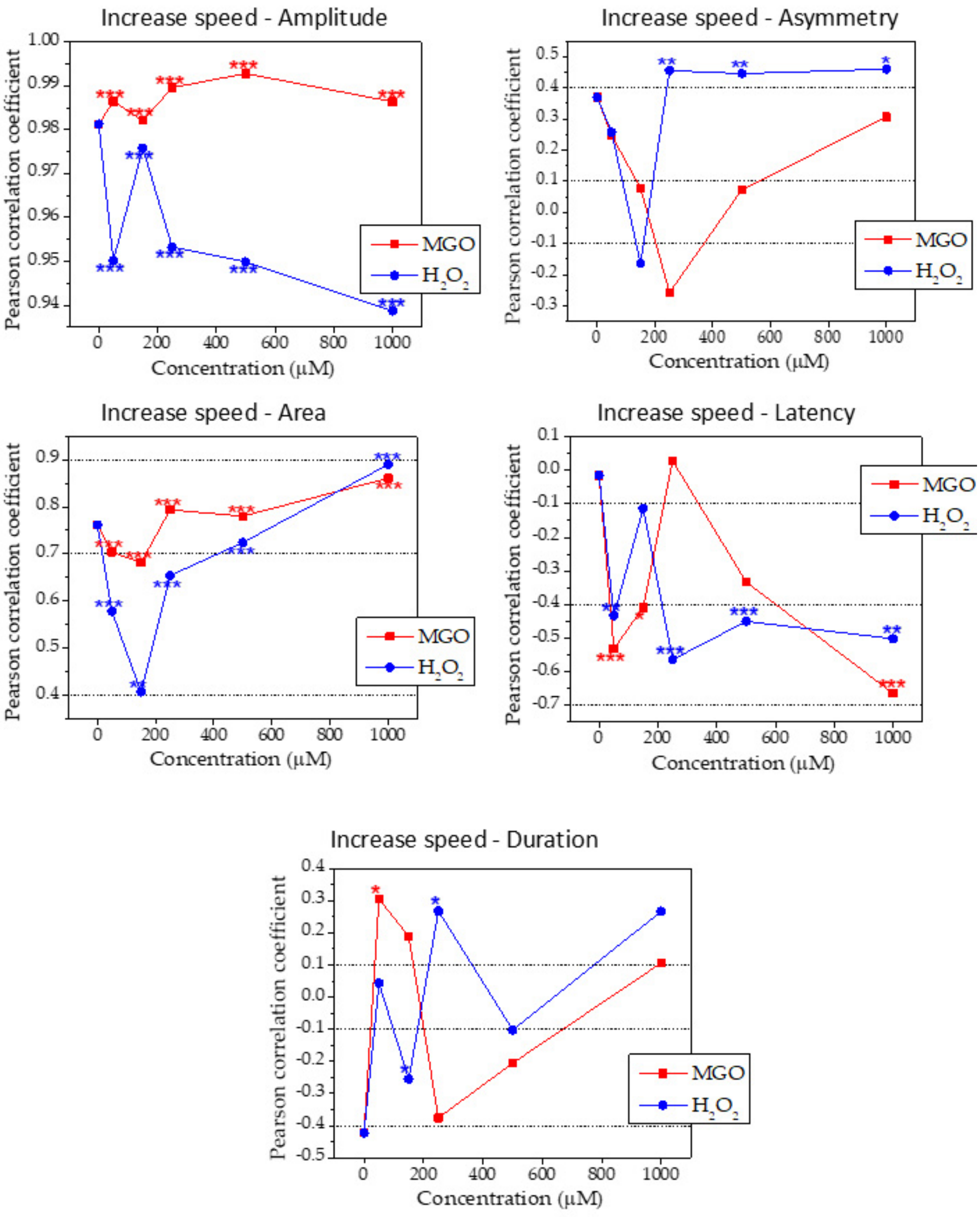

Figure S5. Pearson correlation coefficients between rising velocity and other parameters of the ATP-induced calcium signals. Brain microvascular endothelial cells were exposed for 24 hours to various concentrations of MGO (50, 150, 250, 500, 1000 µM) or H_2O_2 (50, 150, 250, 500, 1000 µM). Statistical analysis was performed using one-way ANOVA, followed by the *post-hoc* Bonferroni test, with significance indicated as $p < 0.05$, $p < 0.01$, $*p < 0.001$.

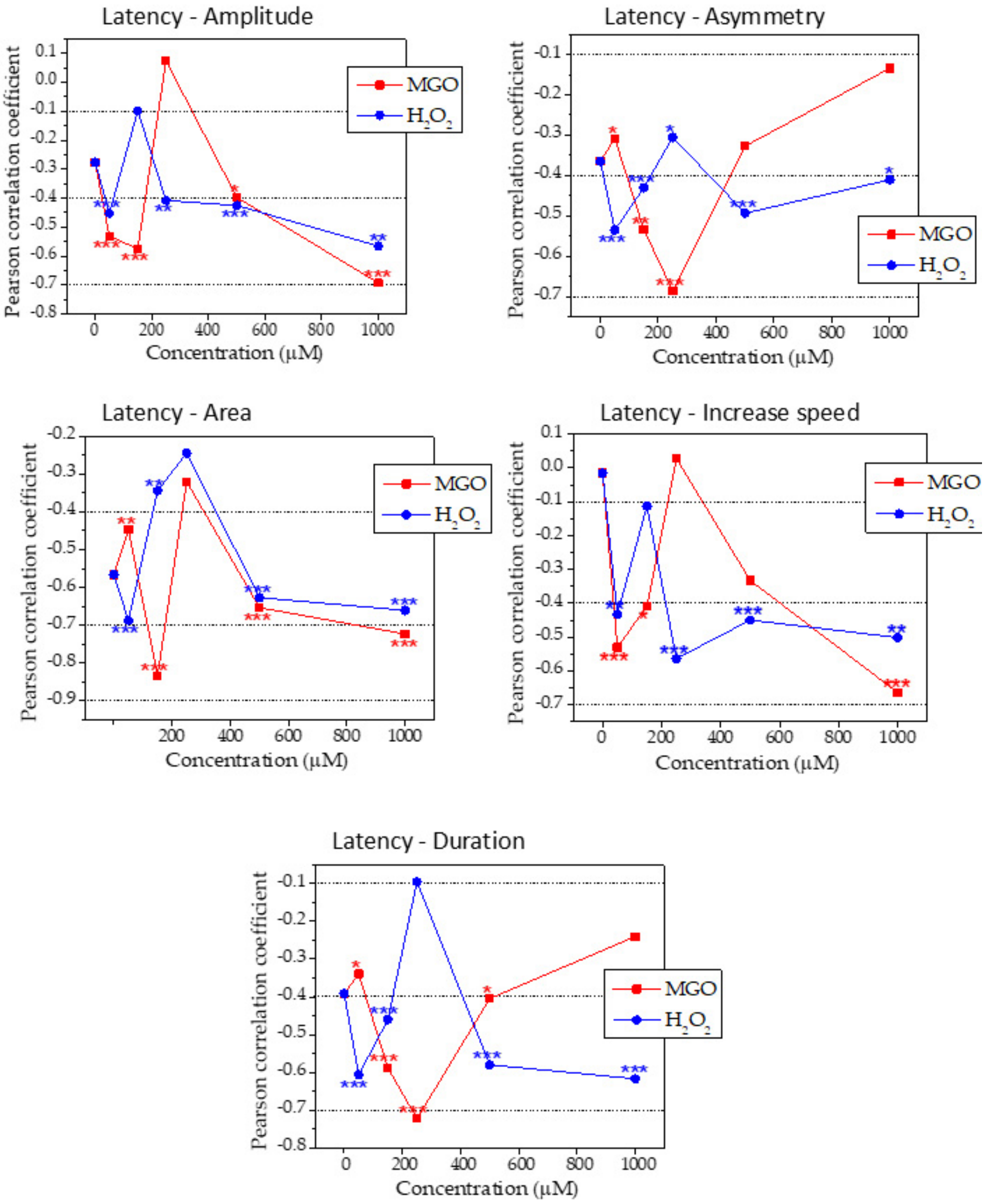

Figure S6. Pearson correlation coefficients between latency and other parameters of the ATP-induced calcium signals. Brain microvascular endothelial cells were exposed for 24 hours to various concentrations of MGO (50, 150, 250, 500, 1000 μM) or H_2O_2 (50, 150, 250, 500, 1000 μM). Statistical analysis was performed using one-way ANOVA, followed by the *post-hoc* Bonferroni test, with significance indicated as $p < 0.05$, $p < 0.01$, *$p < 0.001$.

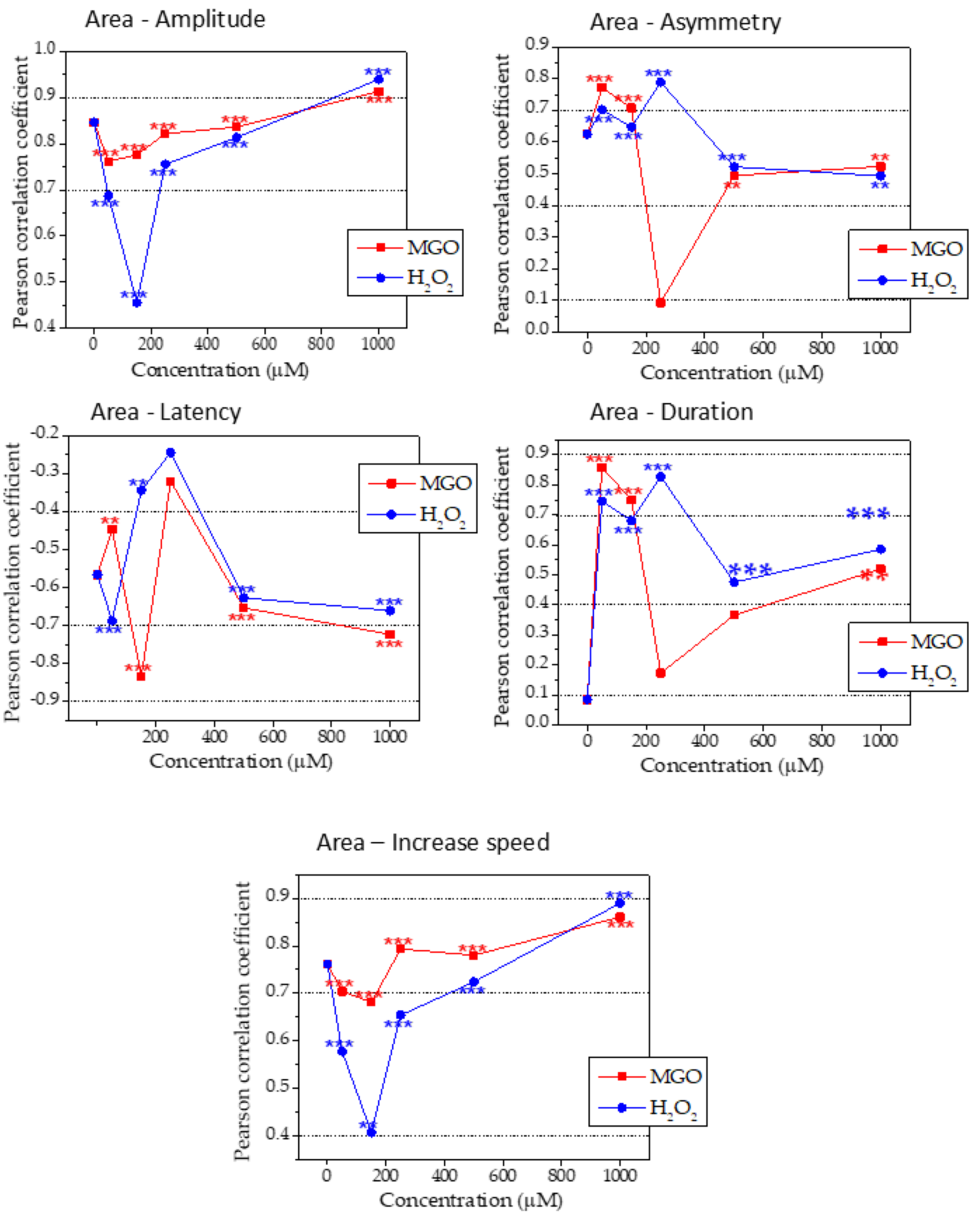

Figure S7. Pearson correlation coefficients between area and other parameters of the ATP-induced calcium signals. Brain microvascular endothelial cells were exposed for 24-h to different concentrations of MGO (50, 150, 250, 500, 1000 μM) or H2O2 (50, 150, 250, 500, 1000 μM). Statistical analysis was done by one-way ANOVA followed by post-hoc Bonferroni test, and indicated as * p<0.05, ** p<0.01, *** p<0.001.

Table S1. Effect of MGO on the Pearson correlation coefficients of calcium signal parameters.

	Area	Amplitude	Latency	Speed	Duration	Asym-metry
Area	1	0.87275	-0.43309	0.36524	0.80519	0.21258
Amplitude	0.87275	1	-0.12402	0.01017	0.59999	-0.21781
Latency	-0.43309	-0.12402	1	-0.50366	-0.4893	-0.55294
Speed	0.36524	0.01017	-0.50366	1	0.74545	0.86226
Duration	0.80519	0.59999	-0.4893	0.74545	1	0.63007
Asymmetry	0.06502	-0.23633	-0.60771	0.71086	0.56483	1

Table S2. Effect of H_2O_2 on the Pearson correlation coefficients of calcium signal parameters.

	Area	Amplitude	Latency	Speed	Duration	Asym-metry
Area	1	0.87516	-0.73109	0.71972	0.58715	-0.07833
Amplitude	0.87516	1	-0.8462	0.94824	0.1754	-0.35211
Latency	-0.73109	-0.8462	1	-0.8487	-0.311128	0.6858
Speed	0.71972	0.94824	-0.8487	1	-0.003881	-0.47812
Duration	0.58715	0.1754	-0.31128	-0.03881	1	0.04319
Asymmetry	-0.07833	-0.35211	0.6858	-0.47812	0.04319	1

To interpret the absolute magnitude of the Pearson correlation coefficient, the following scale was used: 0.00–0.09 (negligible correlation), 0.10–0.39 (weak correlation), 0.40–0.69 (moderate correlation), 0.70–0.89 (strong correlation), and 0.90–1.00 (very strong correlation) [1].

[1] Schober, P.; Boer, C.; Schwarte, L.A. Correlation Coefficients: Appropriate Use and Interpretation. *Anesth. Analg.* **2018**, *126*, 1763–1768. https://doi.org/10.1213/ANE.0000000000002864.

Table S3. Effect of MGO on the Pearson correlations of calcium signal parameters. Only moderate, strong and very strong correlations were indicated.

**Treatment condition**	**Type of correlation**	**Strength of correlation**	**Pair of calcium signal parameters**
MGO	Positive	Very strong	Rising velocity vs Amplitude<br>Duration vs Asymmetry
		Strong	Area vs Amplitude<br>Area vs Asymmetry<br>Duration vs Asymmetry<br>Duration vs Area<br>Area vs Rising velocity
		Moderate	Area vs Asymmetry
	Negative	Strong	Latency vs Area
		Moderate	Latency vs Amplitude<br>Latency vs Asymmetry<br>Latency vs Duration<br>Latency vs Rising velocity<br>Latency vs Area<br>Duration vs Rising velocity

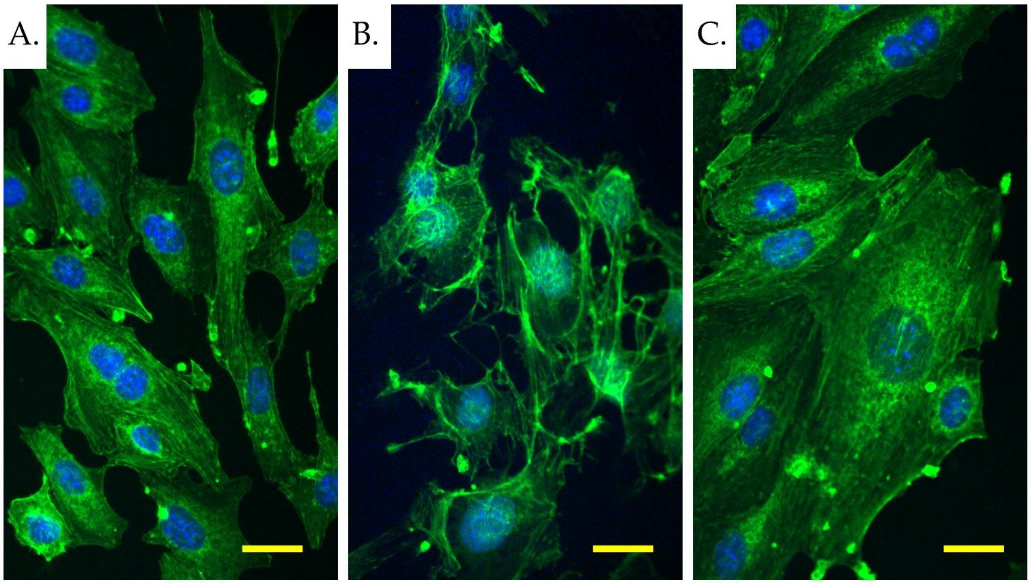

Figure S8. Confocal microscopy images of fluorescently stained brain microvascular endothelial cells. Cells were stained with phalloidin-FITC for the actin filaments (green) and with DAPI for nuclei (blue). Scale 10 μM. (a) Control conditions. (B) 50 μM MGO. (c) 250 μM MGO.

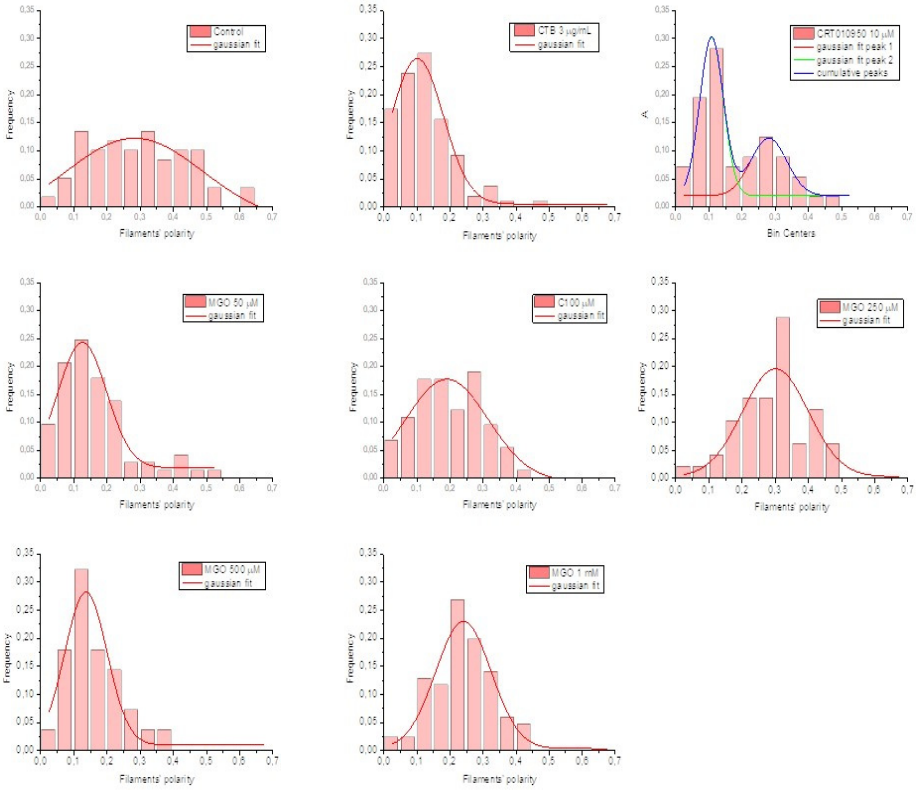

Figure S9. Histograms of the number of cells versus actin filament polarity obtained from confocal microscopy image analysis for each treatment condition. Brain microvascular endothelial cells were fluorescently stained with phalloidin-FITC after exposure to: (a) Control conditions, (b) Positive oxidative control (H_2O_2, 500 µM), (c) Positive cofilin inhibitor (CRT0105950, 10 µM), (d) Positive cytoskeleton remodeling control (CTB, 3 µg/mL), (e-i) MGO (50, 100, 250, 500, 1000 µM). After performing the Fiberscore algorithm analysis on the confocal microscopy images, the number of cells for each treatment condition was plotted against polarity.

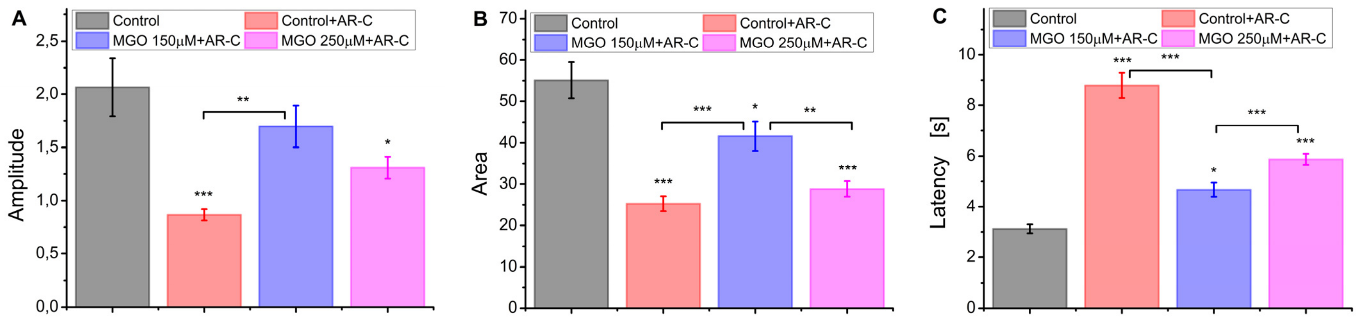

Figure S10: Modulation of calcium transients' parameters: (A) amplitude, (B) area and (C) latency, by MGO in presence of inhibitor AR-C 118925XX (mean ± SE, n: 15-41). Statistical significance was assessed using one-way ANOVA, followed by the Bonferroni post hoc test, and indicated as follows: *$p < 0.05$, **$p < 0.01$, ***$p < 0.001$. Where no other indication, the comparison in the statistical test was done with the Control.
